# A screw-type pacemaker lead implanted in the right atrium perforated the ascending aorta

**DOI:** 10.1186/s43044-024-00494-2

**Published:** 2024-05-24

**Authors:** Tomohiro Nakajima, Yutaka Iba, Tsuyoshi Shibata, Ayumu Osamura, Naoyuki Kamiyama, Ryo Nishikawa, Junji Nakazawa, Nobuyoshi Kawaharada

**Affiliations:** 1 Department of Cardiovascular Surgery, Sapporo Medical University School of Medicine, South-1, West-16, Chuo-Ku, Sapporo, 060-8543 Japan; 2 Department of Cardiovascular, Renal and Metabolic Medicine, Sapporo Medical University School of Medicine, Sapporo, Japan

**Keywords:** Pacemaker lead, Massive bleeding, Cardiac tamponade

## Abstract

**Background:**

Perforation by pacemaker leads, although rare, is a complication reported since the introduction of pacemaker therapy. Although historically reported frequencies were as high as 5%, recent reports have cited frequencies ranging from 1 to 2%. We report a case where a screw-type atrial lead slightly penetrated the right atrial wall, causing chronic abrasion of the ascending aorta, resulting in shock.

**Case presentation:**

A 54-year-old male presented with dilated cardiomyopathy diagnosed at 40 years of age when he developed decompensated heart failure. Despite ongoing treatment, his heart failure worsened, leading to hospitalization at the age of 54. During his hospital stay, he experienced cardiac arrest that required cardiopulmonary resuscitation, followed by a return of spontaneous circulation. He was subsequently transferred to our institution after initiation of venoarterial extracorporeal membrane oxygenation (VA-ECMO) and an intra-aortic balloon pump (IABP). Echocardiography revealed an ejection fraction of 25%, left ventricular end-diastolic diameter of 60 mm, and severe mitral regurgitation (MR). Transcatheter mitral valve repair was performed to treat severe MR, followed by implantation of a cardiac resynchronization therapy defibrillator (CRT-D). Three months later, the patient was brought to our emergency department by ambulance because of hypotension. Contrast-enhanced computed tomography revealed pericardial effusion causing cardiac tamponade, necessitating emergency pericardial decompression via left fourth intercostal mini-thoracotomy and drain placement. Upon transfer to the intensive care unit, 1200 mL of blood was drained from the chest tube, prompting a return to the operating room for a median sternotomy. It was discovered that the pacemaker lead on the left side of the right atrium had slowly eroded into the aorta, leading to perforation. The ascending aorta was repaired and hemostasis was achieved; the patient recovered uneventfully and was discharged on postoperative day 18.

**Conclusions:**

The pacemaker lead perforated the right atrium; chronic abrasion of the lead against the ascending aorta resulted in bleeding from the ascending aorta 3 months later.

## Background

Complications during pacemaker implantation, such as pneumothorax during venous puncture and cardiovascular injury during lead insertion, have been previously reported. Among these, cardiac perforation occurs in approximately 1% of cases and is a rare but serious complication that can lead to cardiac tamponade or death. Injuries to the right atrium and right ventricle are common; however, reports of damage to the ascending aorta are rare. In this case, a screw-type pacemaker lead protruded from the right atrium and subsequently chronically abraded the ascending aorta, ultimately causing perforation and bleeding from the ascending aorta.

## Case presentation

**Fig. 1:**
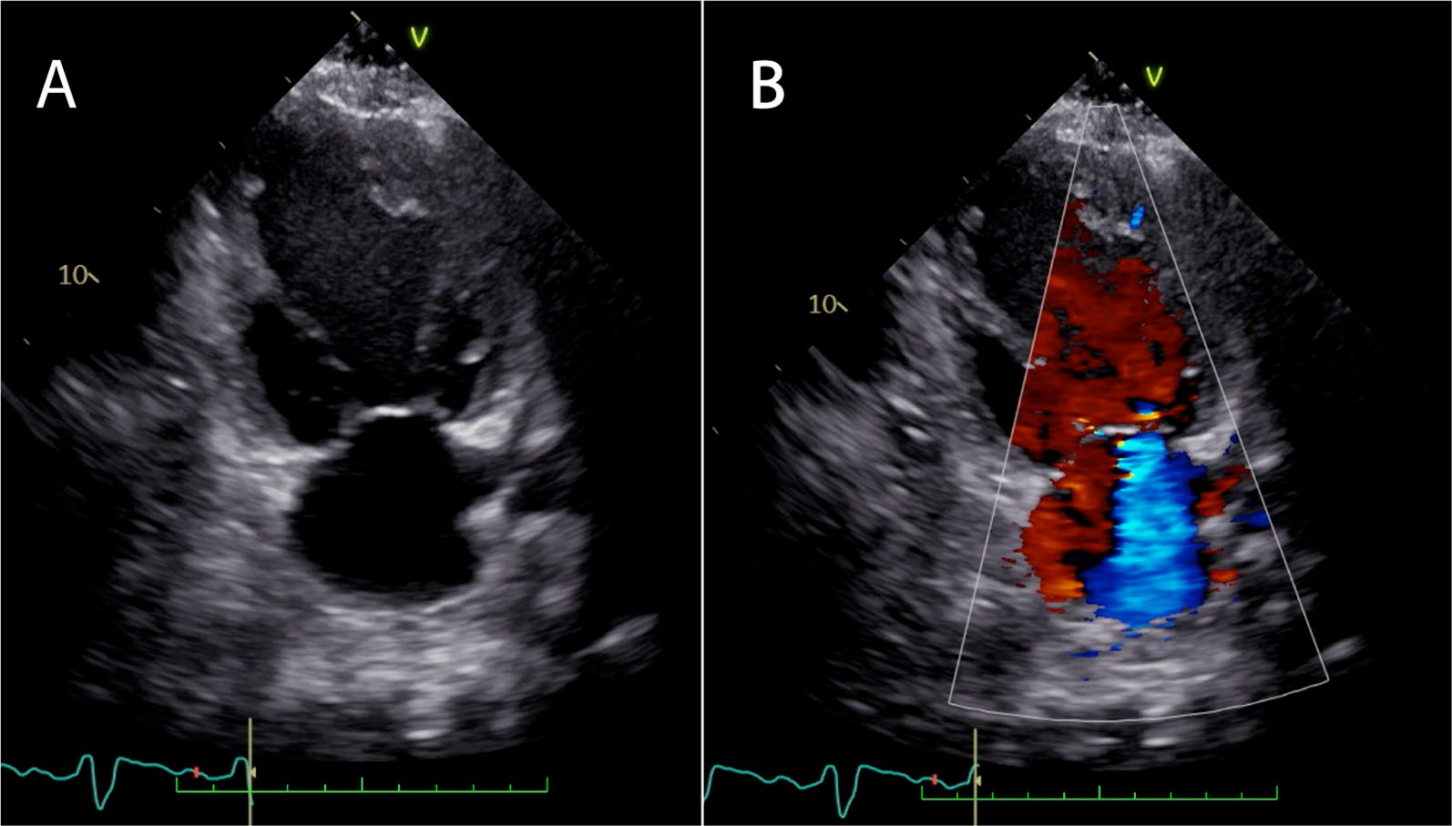
Preoperative echocardiogram. **A** Biplane mode of early systolic phase. **B** Severe mitral valve regurgitation was detected

**Fig. 2:**
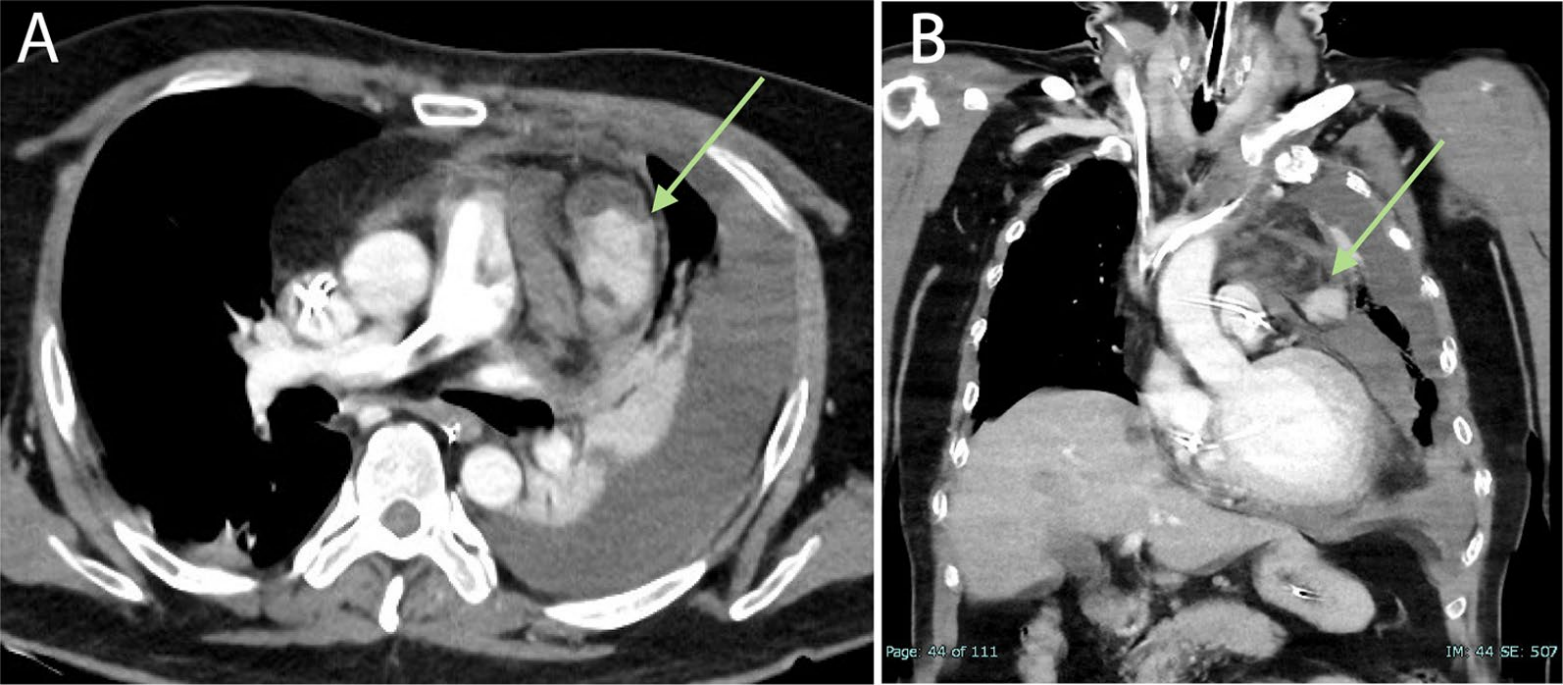
Preoperative images of enhanced computed tomography. **A** Axial view and **B** coronal view. Venous hemorrhage from the thymus gland was noted (green arrow)

**Fig. 3:**
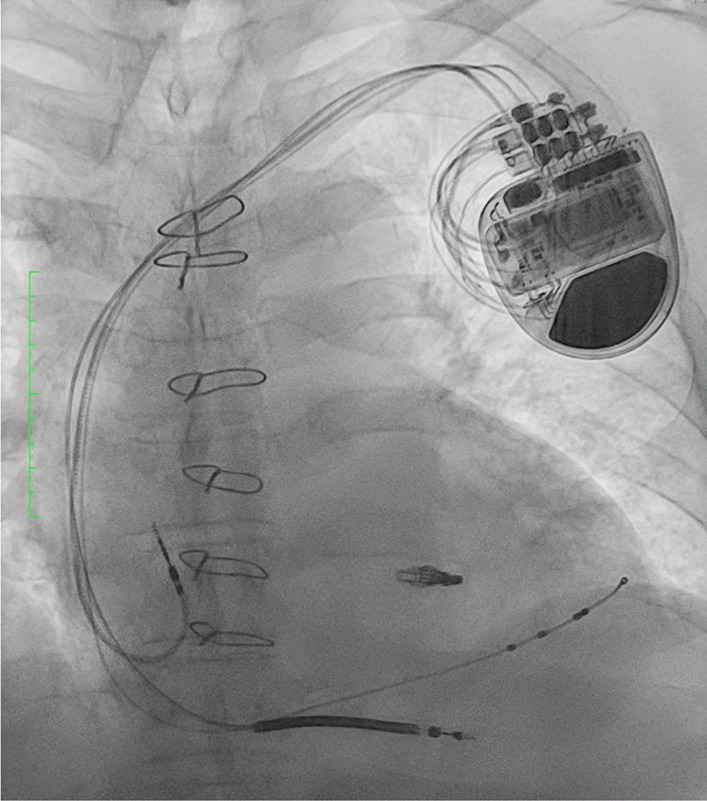
Postoperative chest radiograph after cardiac resynchronization therapy-defibrillator placement

**Fig. 4:**
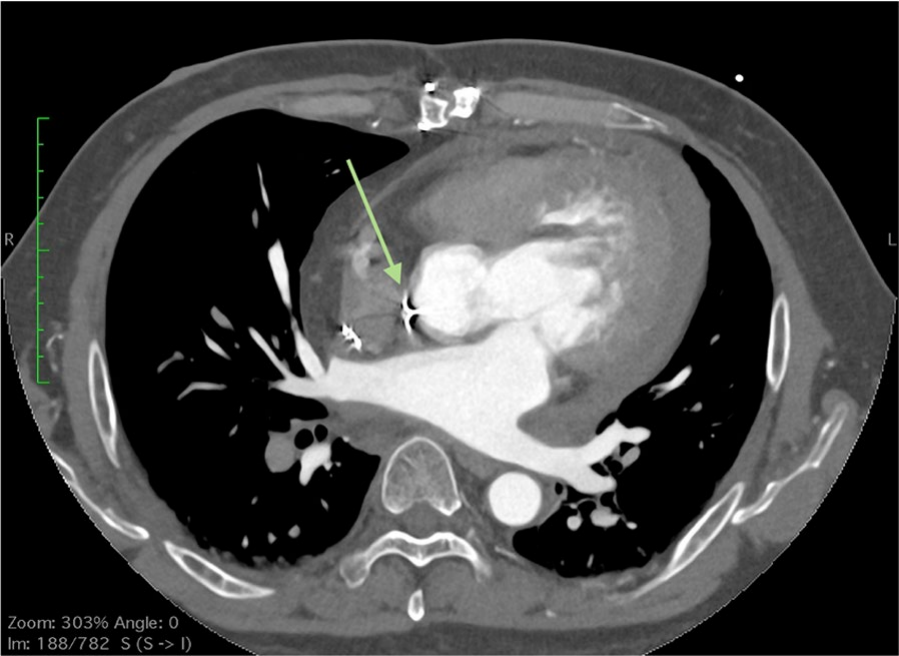
Preoperative computed tomography image to evaluate for cardiogenic shock. A 20-mm pericardial effusion was observed posterior to the left ventricular wall. The atrial lead tip was located proximal to the ascending aorta (green arrow)

**Fig. 5:**
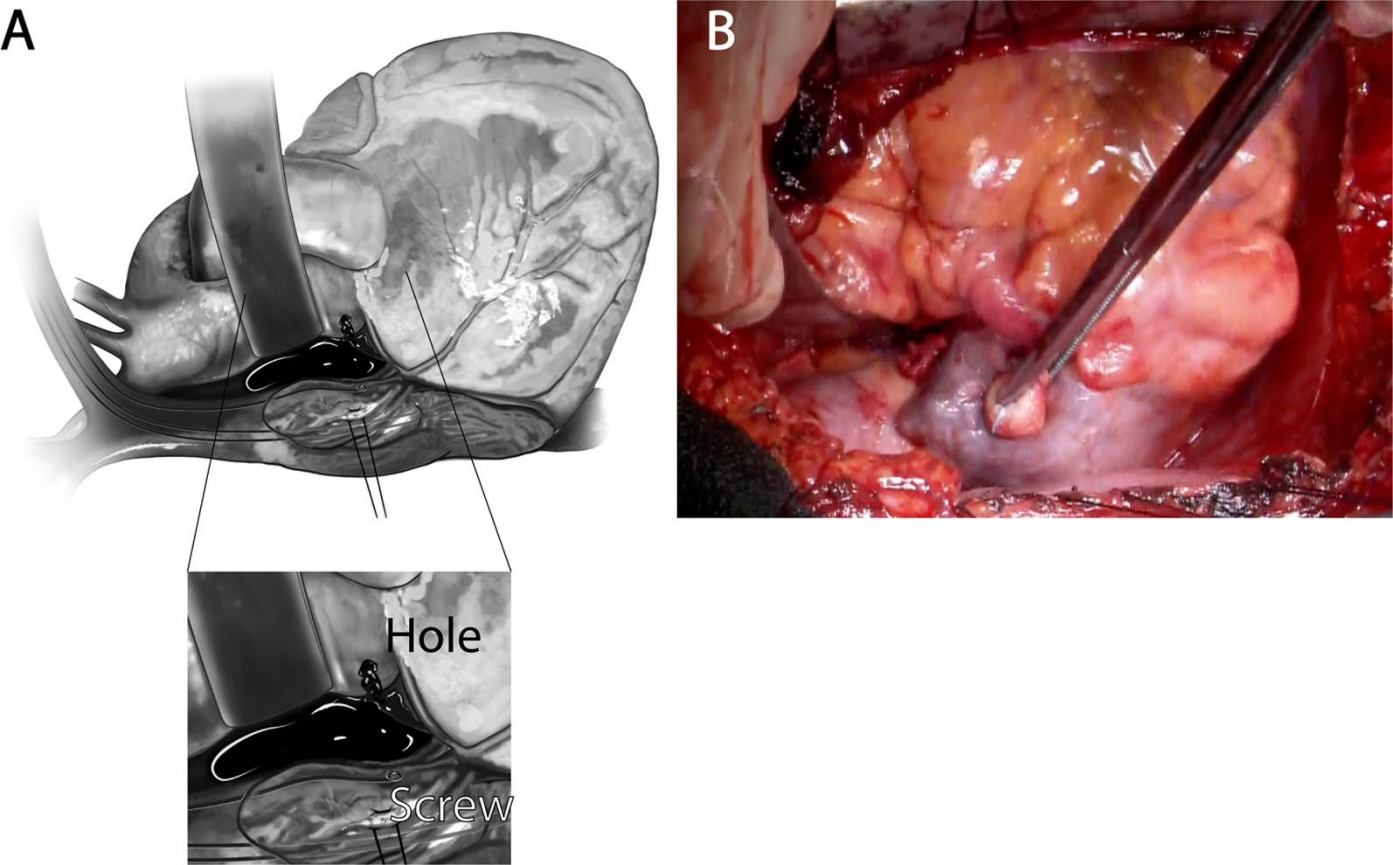
**A** Intraoperative schema. A 2-mm-sized hole was observed in the ascending aorta relative to the tip of an atrial lead penetrating from the right atrium. **B** Right atrium with a pledget to cover the pacemaker lead

The patient was a 54-year-old male who initially developed heart failure at the age of 40 and was diagnosed with dilated cardiomyopathy. A myocardial biopsy was conducted and a diagnosis of idiopathic dilated cardiomyopathy was made. The patient was treated with angiotensin-converting enzyme (ACE) inhibitor, beta-blocker, and sacubitril valsartan. The patient continued the treatment thereafter. At the age of 54, he experienced respiratory distress, leading him to seek medical attention and subsequent hospitalization with the diagnosis of acute exacerbation of chronic heart failure. Catecholamines were administered to treat heart failure. During hospitalization, the patient experienced cardiac arrest and underwent cardiopulmonary resuscitation, including chest compressions, which resulted in the restoration of cardiac activity. Coronary angiography was performed, but there was no significant stenosis, and the cause of cardiac arrest was unknown. Venoarterial extracorporeal membrane oxygenation (VA-ECMO) and intra-aortic balloon pump (IABP) were initiated, and the patient was subsequently transferred to our hospital for multidisciplinary treatment. VA-ECMO was established by securing a blood supply channel from the right femoral artery and a debridement channel from the right femoral vein. Echocardiography revealed an ejection fraction (EF) of 26%, a left ventricular end-diastolic diameter (LVDD) of 64 mm, and severe mitral regurgitation (MR) (Fig. 1A, B). Gradual recovery of cardiac function was observed, and ECMO was discontinued on the third day after transfer. On the fifth day after transfer, the patient developed hypotension. Contrast-enhanced computed tomography (CT) revealed bleeding from the thymus and a significant hematoma in the mediastinum (Fig. 2A, B). An emergency sternotomy was performed for thymic hemostasis and hematoma removal. Subsequently, catecholamine support was discontinued and the patient was weaned off the ventilator. For residual severe MR, transcatheter mitral valve repair was performed on the 84th day after transfer. The mechanism of mitral valve regurgitation in this case was primarily due to the tethering caused by left ventricular dilation and mitral annular enlargement, which is often a result of idiopathic dilated cardiomyopathy. The placement of a single MitraClip successfully controlled the mitral regurgitation. The patient was discharged ambulatory to home on the 92nd postoperative day. One month later, the cardiac resynchronization therapy defibrillator (CRT-D) was implanted. Although a screw-type atrial lead was initially placed, it was repositioned to the free wall of the left atrium because of poor electrical potentials and thresholds in the right atrial appendage (Fig. 3). Three months after CRT-D insertion, the patient was transported to our hospital by ambulance because of hypotension. Contrast-enhanced CT revealed a pericardial effusion of approximately 20 mm adjacent to the posterior wall of the left ventricle (Fig. 4), leading to a diagnosis of cardiac tamponade. Emergency left fourth intercostal mini-thoracotomy was performed for pericardial drainage, and a drain was placed. Upon returning to the ICU, 1200 mL of bleeding was observed from the drain, necessitating a median sternotomy in the operating room. In the previous surgery, the pericardium was not opened because of bleeding from the thymus tissue. When reopening the chest, we were careful to avoid damage to the unnamed vein, and there was no adhesion in the pericardial sac. The pacemaker lead was exposed approximately 1 mm from the left side of the right atrium, likely causing chronic abrasion of the aorta (Fig. 5). Bleeding from the ascending aorta was observed and was subsequently controlled. The hole was closed with 4-0 prolene thread with pledget and hemostasis was achieved. Although the lead screw had protruded from the right atrium, there was no bleeding from the atrium itself. We determined that attempting to re-position the lead could significantly extend the surgical time, thereby increasing the overall invasiveness of the procedure. As a result, our strategy during this operation was to focus solely on hemostasis without attempting to re-position the lead. The protruding area of the right atrial lead was covered with biological glue and the chest was closed. As the surgical procedure progressed, hemostasis was achieved, and before closing the chest, the functionality of the defibrillator was checked, and no issues were observed. Subsequent recovery was uneventful, and the patient was discharged on the 18th postoperative day.

## Discussion

The onset of heart damage due to cardiac pacemakers can vary depending on several factors, including pacemaker type and complications that may arise after implantation [1]. In some cases, heart damage may occur during the implantation procedure itself if there are complications such as perforation of the heart muscle or damage to the surrounding tissues [2]. However, the most common types of heart damage associated with pacemakers occur gradually over time due to their long-term use [3].

Although perforations in the right atrium and ventricle are common, instances affecting the aorta are rare [4]. In this case, the screw-type atrial lead of the CRT-D device was placed on the left side of the right atrium. It is speculated that the thin wall of the right atrium allows the lead to penetrate shortly after insertion or during the early stages, causing the metal to protrude slightly outside the heart. This metal contact with the ascending aorta results in chronic abrasion, gradually wearing down the aortic wall and eventually leading to perforation. As supporting evidence, the perforation in the ascending aorta was conical in shape.

While perforations caused by pacemaker leads typically require removal, in this case, the metal protrusion was approximately 1 mm, and there was adhesion to the surrounding tissue, which increased the risk of bleeding during removal [5]. Therefore, to mitigate this risk, the protruding 1-mm metal was covered with a pledget to prevent direct contact with the aorta. Additionally, felt was applied to the aortic side and tissue glue was inserted between them to secure them in place and prevent future movement.

## Conclusions

A 54-year-old male diagnosed with dilated cardiomyopathy underwent CRT-D implantation. Three months later, he experienced aortic bleeding, leading to shock. Surgical findings revealed that the pacemaker screw-type atrial lead placed on the left side of the right atrium slightly penetrated the right atrial wall. A 2-mm conical hole was observed in the adjacent ascending aorta, where it came into contact with the lead. Chronic abrasion was considered to be the cause of the ascending aortic injury. Hemostasis and padding were performed to prevent further abrasion, and the patient was discharged.

## Data Availability

Not applicable.

## Abbreviations

ECMO: Extracorporeal membrane oxygenation
IABP: Intra-aortic balloon pumping
MR: Mitral valve regurgitation
CRT-D: Cardiac resynchronization therapy-defibrillator
